# Therapeutic Effects of Ceranib-2 and Irinotecan Combination on Colon Cancer

**DOI:** 10.3390/cimb48080841

**Published:** 2026-08-19

**Authors:** Oğuzhan Selvi, Canan Vejselova Sezer, Hüseyin İzgördü, Hatice Mehtap Kutlu

**Affiliations:** 1 Medical Oncology Clinic, Faculty of Medicine, Bezmialem Vakif University, İstanbul 34093, Turkey; selvioguz@gmail.com; 2 Department of Biology, Faculty of Arts and Sciences, Kütahya Dumlupınar University, Kütahya 43100, Turkey; 3 Department of Chemistry, Faculty of Sciences, Eskisehir Osmangazi University, Eskisehir 26040, Turkey; huseyinizgordu@gmail.com; 4 Department of Biology, Faculty of Science, Eskisehir Technical University, Eskisehir 26470, Turkey; hmkutlu@eskisehir.edu.tr

**Keywords:** irinotecan, Ceranib-2, HT-29, proliferation, apoptosis

## Abstract

The present study investigated the anticancer effects of irinotecan (IR) combined with the acid ceramidase inhibitor Ceranib-2 in HT-29 human colorectal cancer cells. Cell viability was assessed using the MTT assay, apoptosis was evaluated by Annexin V flow cytometry, and morphological alterations were examined by fluorescence microscopy. Both irinotecan and Ceranib-2 significantly reduced cell viability in a dose-dependent manner. Notably, the combination treatment produced greater growth inhibition than either agent alone (*p* < 0.01). Morphological analysis revealed pronounced apoptotic features, including nuclear condensation, membrane blebbing, and cytoskeletal disruption, particularly in the combination-treated cells. Annexin V analysis further demonstrated that combined treatment markedly increased apoptotic cell populations compared with single-agent treatments (*p* < 0.01). These findings suggest that Ceranib-2 enhances the anticancer activity of irinotecan and that this combination may represent a promising therapeutic strategy for colorectal cancer.

## 1. Introduction

Colorectal cancer (CRC) is the third most common type of cancer worldwide after lung and breast cancer. It ranks fourth in cancer deaths after lung, stomach, and liver cancer. Sphingolipids are bioactive molecules that form the biological membrane structure [1]. The disease arises from gene mutations and epigenetic alterations that transform colonic epithelial cells into colon adenocarcinoma cells. Colon cancer occupies an important place among tumor formations in the gastrointestinal tract worldwide. Although diagnosis at an early stage increases treatability, in the case of metastatic colon cancer, treatment options are limited, and the prognosis worsens [2].

Ceramide and sphingosine of sphingolipids have attracted attention in recent years as mediator molecules that play a critical role in signal transduction and regulation of cancer cell growth processes. Recent studies have shown that ceramide triggers cell growth, proliferation, and cell death in cancer cells. Disruption of sphingolipid metabolism is an important factor in the development of various cancer types and affects cellular growth and metastasis processes. Ceranib-2 is a ceramidase inhibitor that determines the apoptotic process and plays an important role in anticancer processes such as cell cycle arrest and induction of apoptosis [3]. Disruption of sphingolipid metabolism plays a critical role in the development and progression of various types of cancer. This mechanism is emerging as an alternative pathway targeted in cancer therapy. Ceramide, one of the two most important members of sphingolipid metabolism, has pro-apoptotic properties, while sphingosine-1-phosphate (S1P) has anti-apoptotic properties. There is a balance between S1P and ceramide, and in this balance, called the sphingolipid rheostat, the increase in the amount of S1P in the cell is important in cancer formation, such as suppressing ceramide-mediated apoptosis. Therefore, targeting the balance between S1P/ceramide and changing it in favor of ceramide is important for the development of therapeutic strategies in cancer treatments [4,5,6,7,8,9]. Ceranib-2, as a ceramidase inhibitor, breaks down ceramide into the more biologically active S1P. It can induce cell death apoptosis and suppress the growth of cancer cells by increasing intracellular ceramide levels [10]. However, the exact mechanism of action and mediators in which types of cancers are still unknown.

Irinotecan is a chemotherapeutic, especially widely used in the treatment of advanced colorectal cancer. Irinotecan is a precursor drug that must be converted by carboxylesterases into its active form, SN-38 [11]. Camptothecin and its derivatives selectively inhibit eukaryotic DNA topoisomerase I, an essential enzyme involved in processes like replication, transcription, and repair [12]. These agents disrupt DNA-topoisomerase I cleavable complexes, stabilizing them and causing DNA damage through single-strand breaks. During the S phase, collisions with replication forks convert these single-strand breaks into double-strand breaks [13], leading to cell death. Irinotecan is generally used in combination therapies with other chemotherapy drugs in chemotherapy regimens.

Accumulating evidence suggests that resistance to chemotherapy is partly associated with dysregulation of sphingolipid metabolism and reduced ceramide-mediated apoptosis. Since IR induces DNA damage through topoisomerase I inhibition, while Ceranib-2 increases intracellular ceramide levels by blocking acid ceramidase activity, the simultaneous targeting of these two distinct pathways may enhance cancer cell death. Therefore, combining IR with Ceranib-2 may represent a promising therapeutic strategy to improve apoptotic responses and overcome limitations associated with conventional chemotherapy.

In this study, the effects of the combination of Ceranib-2 and irinotecan were investigated at the molecular level. The aim of the study was to evaluate the potential anticancer effect of the combination of these two agents and to demonstrate the therapeutic effects of combination therapy on colon cancer cells. The primary objective of this study was to evaluate whether inhibition of acid ceramidase by Ceranib-2 enhances the anticancer activity in HT-29 colorectal cancer cells when applied together with irinotecan. Specifically, to determine whether Ceranib-2 can potentiate irinotecan-mediated cell death, the effects of Ceranib-2 and IR separately and in combination on cell viability, apoptosis induction, and morphological changes were investigated.

## 2. Materials and Methods

### 2.1. Culture of HT-29 Cells

HT-29 (ATCC^®^ HTB-38™) cells were obtained from ATCC (Manassas, VA, USA). The cells were incubated in Roswell Park Memorial Institute (RPMI-1640) (Sigma-Aldrich, St. Louis, MO, USA) medium containing 10% serum (Fetal Bovine Serum/FBS) (Sigma-Aldrich, St. Louis, MO, USA) and 1% penicillin/streptomycin (Sigma-Aldrich, St. Louis, MO, USA) at 37 °C in an incubator containing 5% CO_2_ and appropriate humidity. Cells were monitored daily and subcultured when they reached approximately 80–90% confluence. For passaging, cells were washed with phosphate-buffered saline (PBS) (Sigma-Aldrich, St. Louis, MO, USA), harvested using 0.25% trypsin-EDTA (Sigma-Aldrich, St. Louis, MO, USA) solution, neutralized with complete culture medium, and centrifuged at 1200 rpm for 5 min. The cell pellet was resuspended in fresh medium and seeded into new culture flasks at an appropriate split ratio. Cells at passage number 15 were used for all experiments [14].

### 2.2. Determination of Cytotoxic Effects in HT-29 Cells by MTT Colorimetric Assay

HT-29 cells were seeded in 96-well cell culture plates with 5 × 10^3^ cells/well. Ceranib-2 (100–3.125 µM) and irinotecan (20–0.625 mg/mL) were added to each well except the controls by serial dilution and were incubated at 37 °C and 5% CO_2_ for 24 h. At the end of the incubation period, 20 μL of MTT dye (5 mg/mL) was added and further incubated at 37 °C for 4 h. At the end, the liquid in the plate was drained, 200 μL DMSO/well was added and the plates were read at 570 nm wavelength on a plate reader (HTX Synergy, Bio-Tek, Winooski, VT, USA). From the absorbances obtained, viability values (%) were calculated compared to the control group. IC_50_ concentrations of Ceranib-2 and IR on HT-29 cells were calculated from the viability percentages. GraphPad 6.0 was used to determine the standard deviations and statistical significance of the values.

### 2.3. Observation of Morphological Changes

Using fluorescent microscopy, the morphological changes in Ceranib-2 and IR on HT-29 cells were examined. In the preparation stage of HT-29 cells to be examined under a fluorescent microscope, firstly, the cells were incubated in 6-well plates with a cell density of 3 × 10^5^ cells in each well were incubated with IC_50_ concentrations of Ceranib-2 and Irinotecan separately and with combined IC_50_ values for 24 h. After 24 h of incubation, the medium was removed, the cells were washed with phosphate buffer (PBS, Sigma-Aldrich, St. Louis, MO, USA), and fixed with glutaraldehyde (Sigma-Aldrich, St. Louis, MO, USA). After fixation, the cells were washed again with PBS, and the morphological changes in the cells were examined by double staining using acridine orange (Sigma-Aldrich, St. Louis, MO, USA) and phalloidin (Sigma-Aldrich, St. Louis, MO, USA) and imaged using a Leica DM 6000B fluorescent microscope (Leica Microsystems, Wetzlar, Germany) [15].

### 2.4. Annexin-V Analysis

For Annexin-V analysis, HT-29 cells were seeded in 6-well plates (5 × 10^5^/well). These cells were incubated with the IC_50_ concentrations of Ceranib-2 and Irinotecan and the IC_50_ concentration of each for combination treatment in an incubator at 37 °C with 5% carbon dioxide for 24 h. Incubated cells were harvested with trypsin and washed in PBS. After washing, 100 μL of cell sample and 100 μL of annexin reagent were added to the flow cytometry tube and kept in the dark at room temperature for 15 min. Samples were analyzed using a cell analyser (Muse TM Cell Analyser Merck, Millipore, Hayward, CA, USA) according to the protocols of the Muse Annexin V kit [16].

### 2.5. Statistical Analysis

All experiments were performed in triplicate and repeated independently three times. Data are presented as mean ± standard deviation (SD). Statistical analyses were performed using GraphPad Prism version 6.0 (GraphPad Software, San Diego, CA, USA). Differences among groups were analyzed using one-way analysis of variance (ANOVA) followed by Tukey’s multiple comparison test. A value of *p* < 0.05 was considered statistically significant.

## 3. Results

### 3.1. MTT Cytotoxicity Test Results

**Figure 1:**
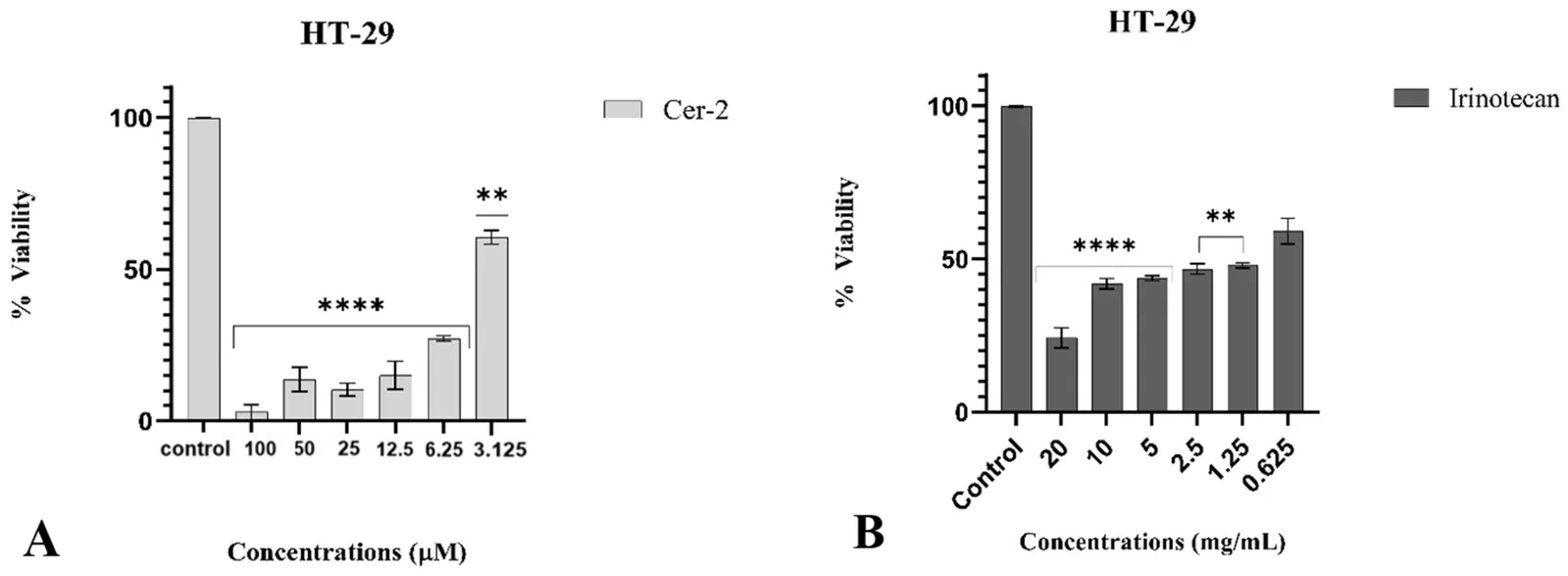
Effects of Ceranib-2 (**A**) and irinotecan (**B**) on the viability of HT-29 colorectal cancer cells after 24 h of treatment, as determined by the MTT assay. Data are presented as the mean ± SD of three independent biological experiments (*n* = 3). Statistical significance was analyzed using one-way ANOVA followed by Tukey’s multiple-comparisons test. ** *p* < 0.01, **** *p* < 0.001.

Ceranib-2 (Cer-2) exhibited a pronounced dose-dependent cytotoxic effect on HT-29 colorectal cancer cells. At higher concentrations (100–25 µM), cell viability dropped below 10%, showing a highly significant reduction compared to the control group. At lower concentrations (6.75–3.375 µM), a partial recovery in cell viability was observed, with a notable increase at 3.375 µM (Figure 1A). These findings demonstrate that Cer-2 exerts strong antiproliferative activity in HT-29 cells, and that its effect is clearly concentration-dependent. Irinotecan demonstrated a clear concentration-dependent cytotoxic effect on HT-29 colorectal cancer cells. At higher concentrations (20–10 mg/mL), cell viability was markedly reduced and showed a highly significant decrease compared to the control group. Intermediate doses (5–2.5 mg/mL) resulted in moderate reductions in viability, with statistically significant differences. At the lowest tested concentration (0.625 mg/mL), cell survival partially recovered, indicating diminished cytotoxicity (Figure 1B). These findings confirm that irinotecan exerts strong antiproliferative activity on HT-29 cells, with its effect diminishing progressively at lower concentrations. Both Cer-2 and irinotecan demonstrated substantial antiproliferative activity against HT-29 colorectal cancer cells, indicating that each compound possesses potent cytotoxic potential and may contribute favorably to therapeutic strategies targeting colorectal malignancies (Table 1).

### 3.2. Results of Morphological Analysis

**Figure 2:**
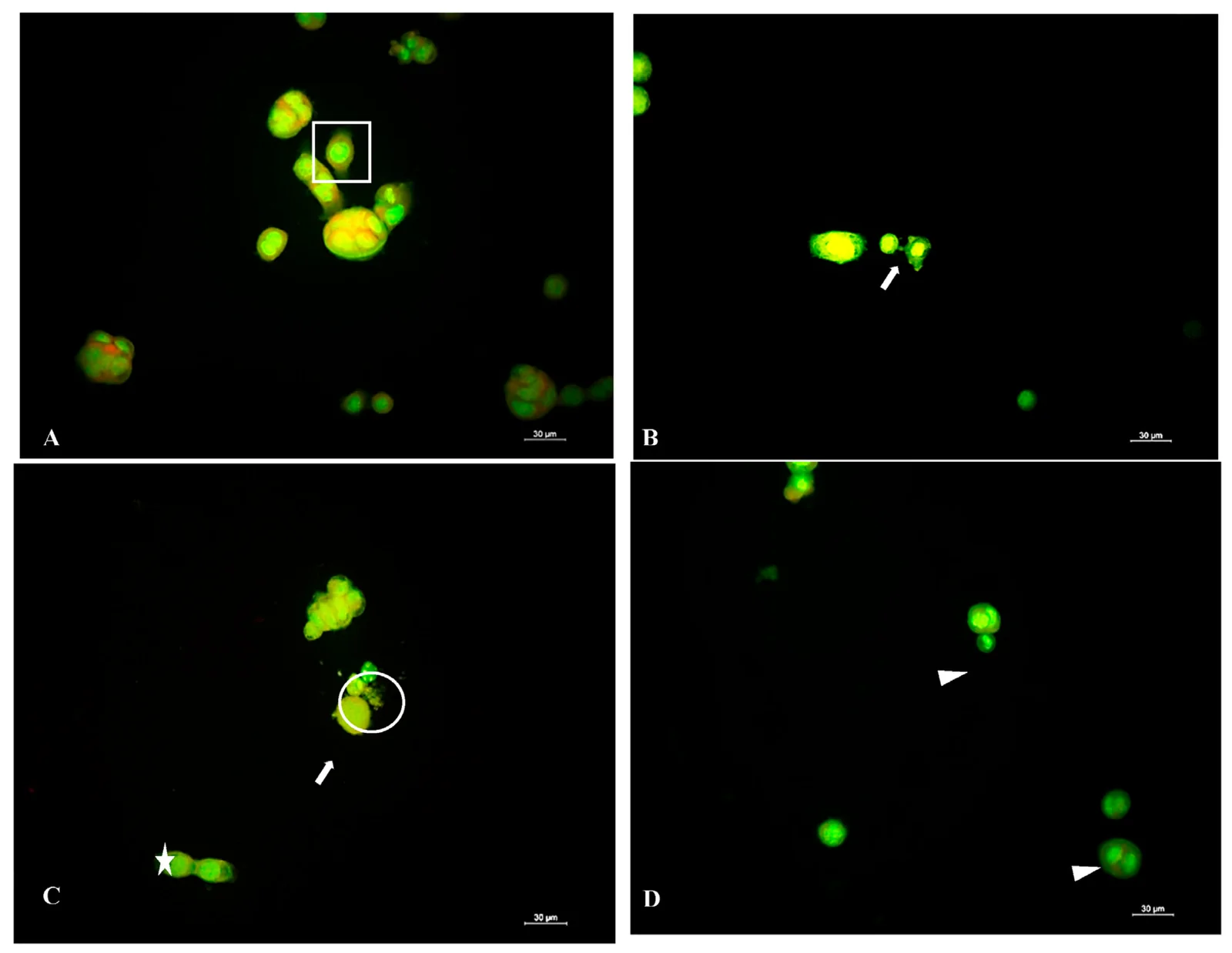
Representative fluorescence microscopy images of HT-29 cells following 24 h of treatment: untreated control (**A**), Ceranib-2-treated (**B**), irinotecan-treated (**C**), and Ceranib-2/irinotecan combination-treated cells (**D**). Morphological changes associated with apoptosis are indicated as follows: rectangle, compact nucleus and condensed cytoskeleton; arrow, plasma membrane blebbs; circle, fragmented nuclei; asterisk, cytoskeletal disruption (holes in the cytoskeleton); and arrowhead, horseshoe-shaped nucleus. Scale bar = 30 µm. Representative images from three independent biological experiments are shown. Green color-nuclei, Red color-cytoskeleton.

**Figure 3:**
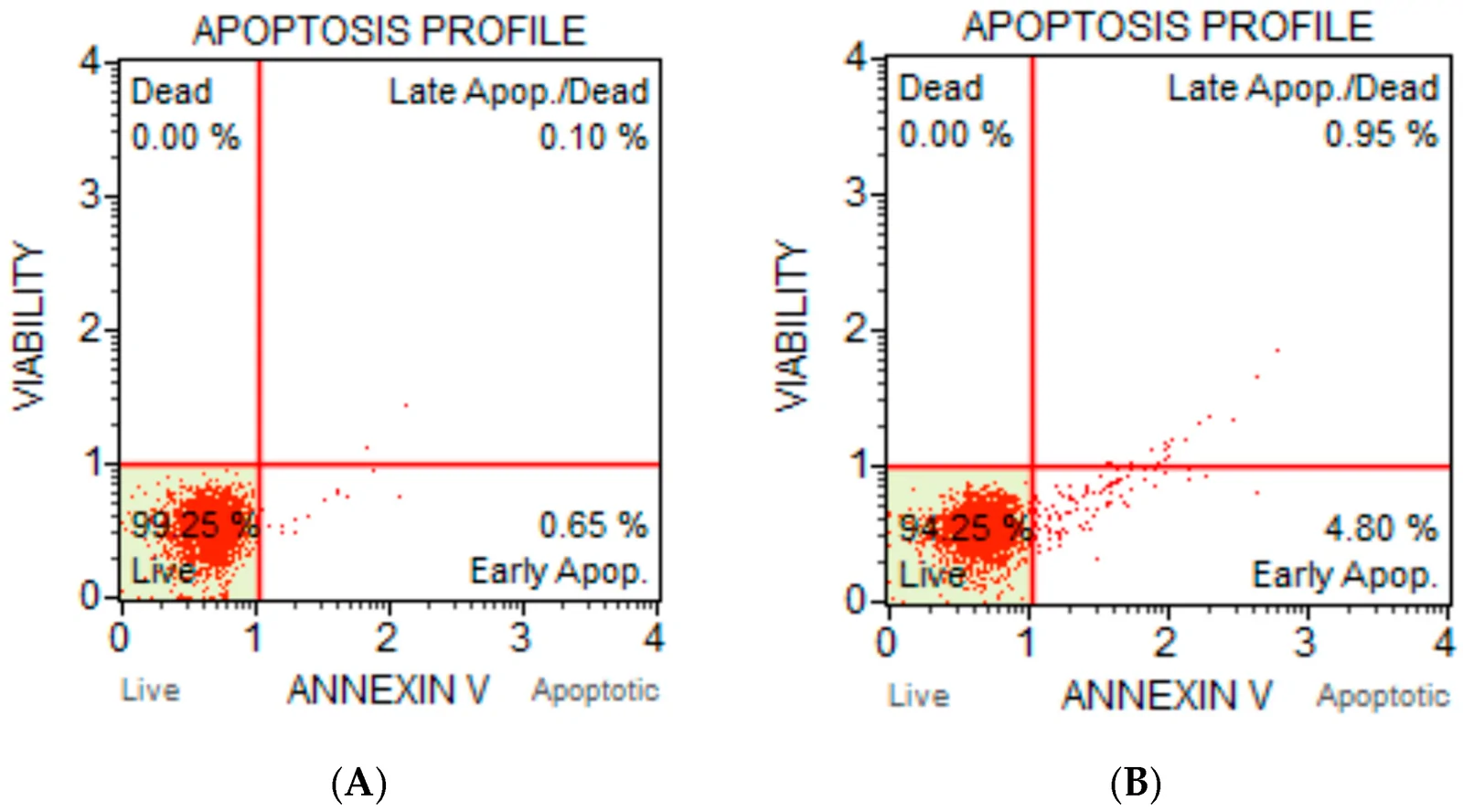

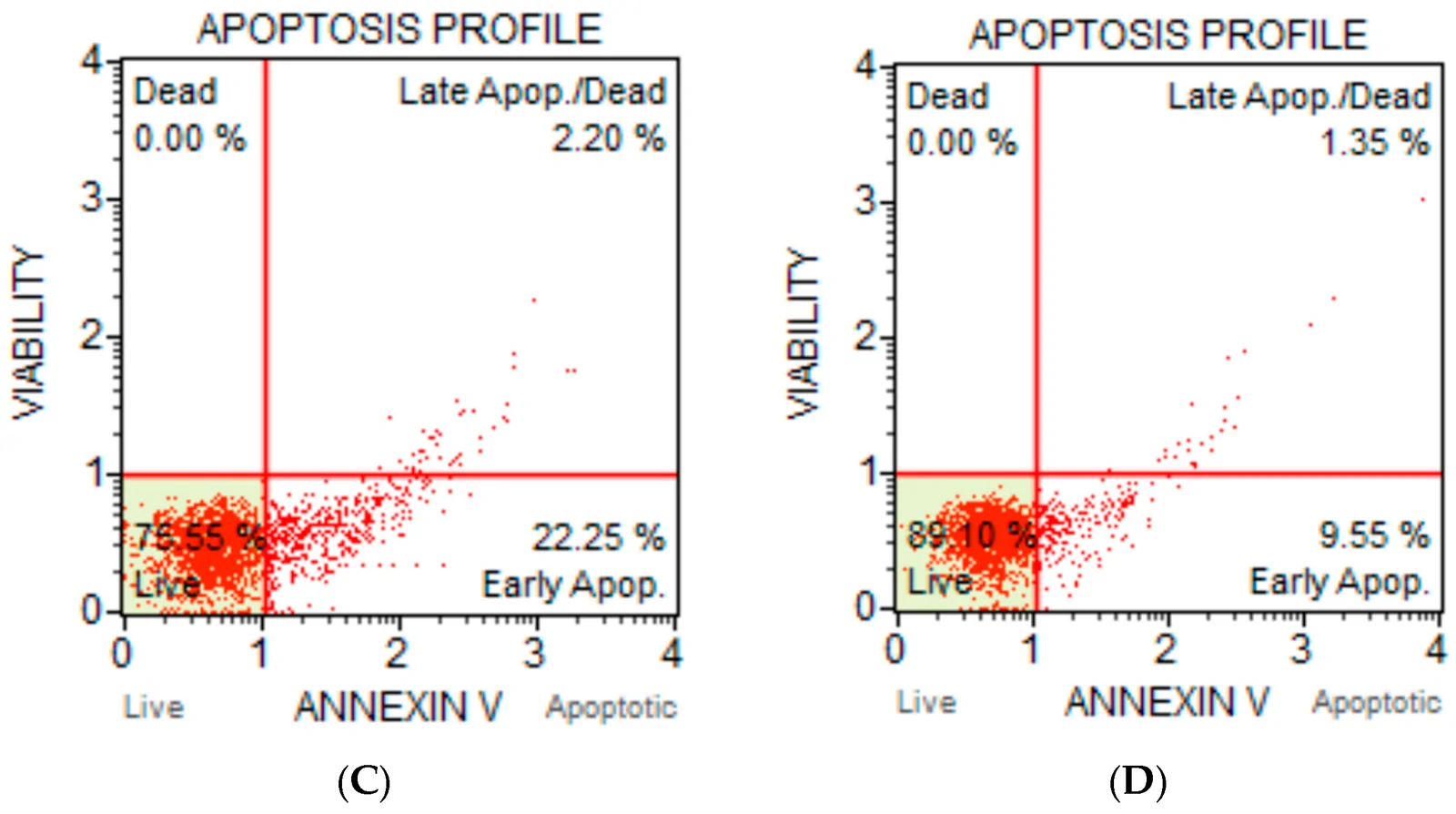
Representative Annexin V flow cytometry analysis of apoptosis in HT-29 cells following 24 h of treatment: untreated control (**A**), irinotecan-treated (**B**), Ceranib-2-treated (**C**), and Ceranib-2/irinotecan combination-treated cells (**D**). Representative dot plots from three independent biological experiments are shown. Red dots: Analysed population of cells; Green color: Area of live cell population.

Fluorescence microscopy revealed distinct morphological alterations associated with apoptosis in HT-29 cells following Ceranib-2, irinotecan, and combined treatment (Figure 2). While untreated cells (Figure 2A) displayed intact nuclei and well-preserved cytoskeletal organization, Ceranib-2 exposure led to prominent membrane blebbing and nuclear condensation (Figure 2B). Irinotecan treatment resulted in fragmented nuclei and cytoskeletal disruption, indicating advanced apoptotic damage (Figure 3C). Notably, the combined Ceranib-2-irinotecan treatment intensified these features, including the appearance of horseshoe-shaped nuclei and pronounced cytoskeletal gaps, suggesting a synergistic enhancement of apoptotic cell death (Figure 3D). These observations support the cytotoxic and apoptosis-inducing potential of both agents, particularly when used in combination.

### 3.3. Flow Cytometry Results

Annexin V flow cytometry analysis revealed a clear increase in apoptotic cell populations following treatment with Ceranib-2, irinotecan, and especially their combined exposure in HT-29 cells (Figure 3). Untreated control cells displayed a minimal apoptotic profile, with nearly all cells remaining within the viable quadrant. The percentage of viable cells was found to be 99.25%, 94.25%, 89.25%, and 75.55% in untreated HT-29 cells (Figure 3A) and cells treated with irinotecan (Figure 3B), ceranib-2 (Figure 3C), and their combination (Figure 3D), respectively. The total apoptotic cell percentages were 0.75%, 5.75%, 10.9% and 24.25% in the untreated cells and cells exposed to irinotecan, ceranib-2 and their combination for 24 h. Early apoptotic cells were 0.65%, 4.80%, 9.55% and 22.25% respectively in control cells and those exposed to irinotecan, ceranib-2 and the irinotecan-ceranib-2 combination.

**Figure 4:**
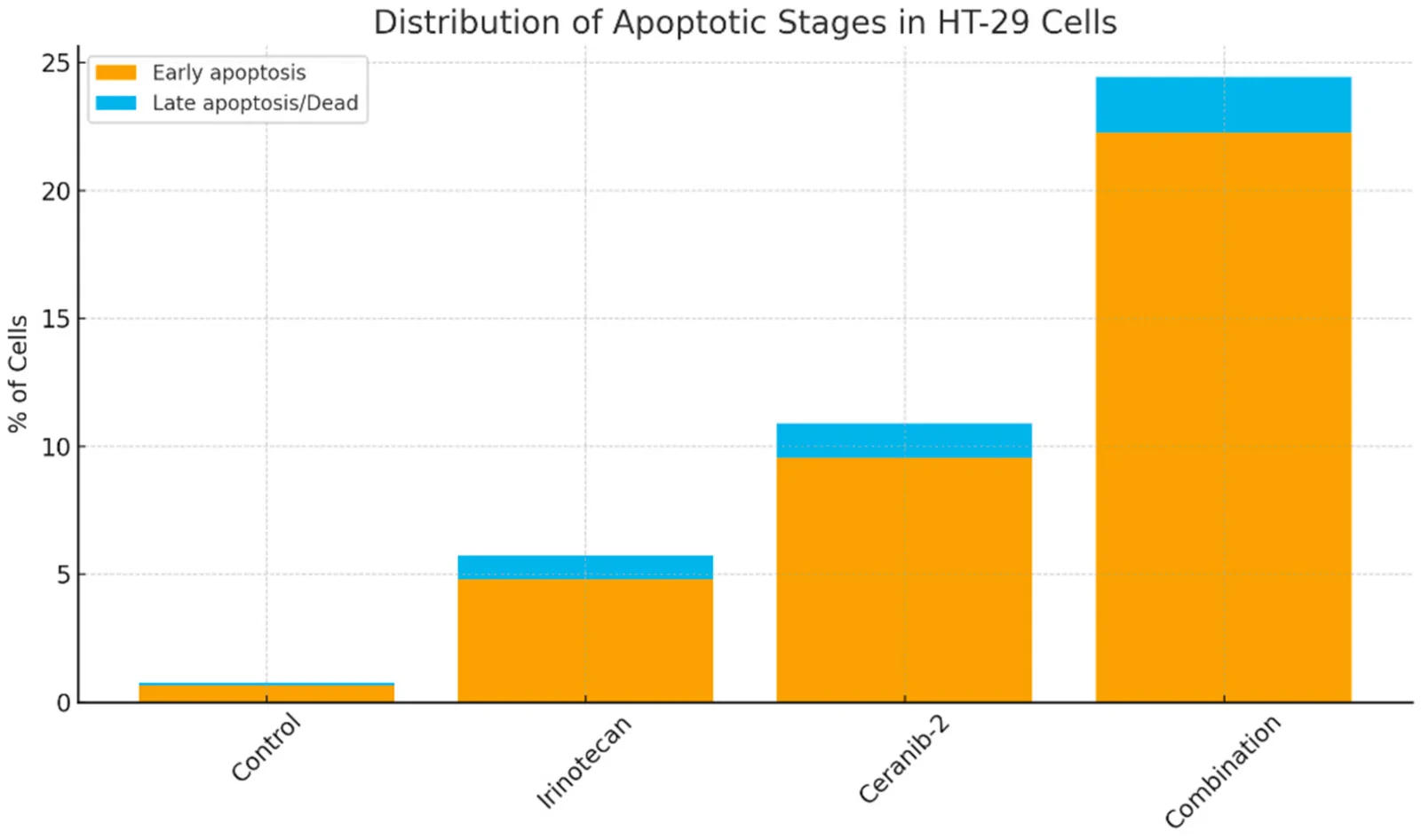
Quantitative distribution of early apoptotic and late apoptotic/dead HT-29 cells following 24 h of treatment with irinotecan, Ceranib-2, or their combination, as determined by Annexin V technique. Data are presented as the mean ± SD of three independent biological experiments (*n* = 3).

**Figure 5:**
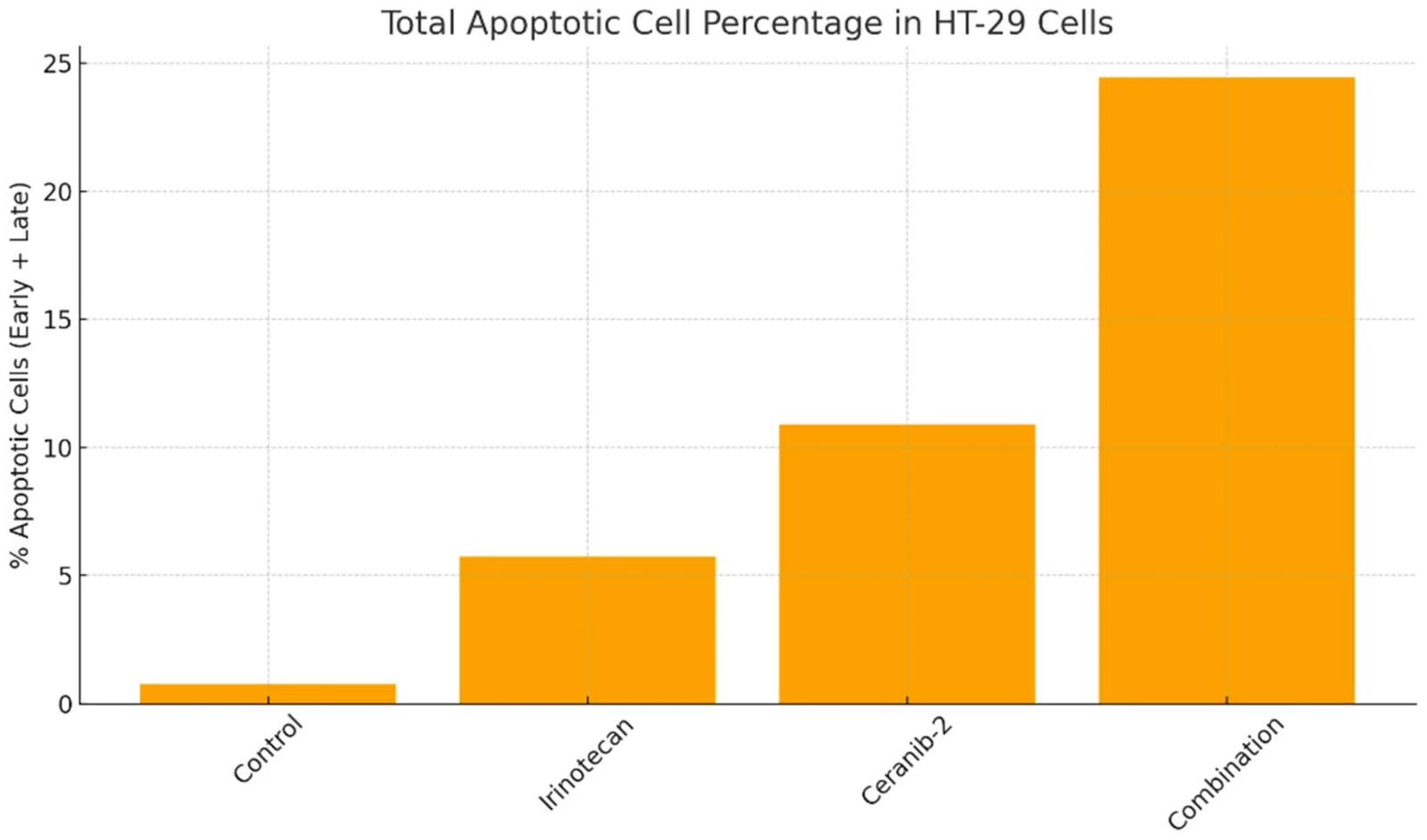
Total apoptotic cell percentage in HT-29 cells following 24 h of treatment with irinotecan, Ceranib-2, or their combination, as determined by Annexin V technique. Total apoptosis was calculated as the sum of early and late apoptotic cell populations. Data are presented as the mean ± SD of three independent biological experiments (*n* = 3).

Stacked bar plots illustrate the relative proportions of early and late apoptotic cell populations obtained from Annexin V analysis (Figure 4). Irinotecan and Ceranib-2 each increased early apoptosis compared to the untreated control, with Ceranib-2 exhibiting a stronger apoptotic induction. The combined treatment resulted in the highest levels of both early and late apoptosis, indicating an amplified apoptotic response when both compounds were applied concurrently (Figure 5). Data represent the percentage of cells in each apoptotic stage based on quadrant distribution.

Apoptosis was quantified based on Annexin V analysis, and total apoptotic rate was calculated as the sum of early and late apoptotic cell populations. Both agents individually increased apoptosis relative to the untreated control, with Ceranib-2 inducing a stronger apoptotic response compared to irinotecan. The combined treatment resulted in the highest apoptotic ratio, indicating an enhanced pro-apoptotic effect when both compounds were applied together. Data are presented as mean percentages derived from the corresponding Annexin V quadrant distributions.

## 4. Discussion

This study aimed to investigate the in vitro effects of Ceranib-2, an acid ceramidase inhibitor, and IR, a topoisomerase I inhibitor widely used for CRC treatment, on HT-29 colorectal cancer cells. To investigate the role of Ceranib-2 and Irinotecan in the growth of HT-29, human colorectal adenocarcinoma cells with epithelial morphology. HT-29 cells are sensitive to the chemotherapeutic drugs 5-fluorouracil and oxaliplatin [17]. According to the results, the IC_50_ value of Irinotecan in HT-29 cells was found as 5.4 µΜ and Ceranib-2 as 4.1 µM for 24 h. It was observed that the Ceranib-2 and IR combination had an antiproliferative effect in HT-29 cells at low concentrations depending on the applied concentration and time, but showed more effective cytotoxicity in combination compared to single exposure. The combination of Ceranib-2 and IR caused morphological changes in HT-29 cells at 24 h, indicating a pro-apoptotic effect. We have shown that inhibition of acid ceramidase induced an increase in ceramide levels and apoptosis in colon cancer cells, as well as a decrease in cell growth. In terms of phosphatidylserine localization, which is an apoptotic marker, it was found that the combination of Ceranib-2 and IR increased the translocation of phosphatidylserine in HT-29 cells and increased the number of cells in early and late apoptosis compared to Ceranib-2 and IR treated alone. The IC_50_ value of IR for HT-29 cells was found to be 5.17 µM in the study by Valérie Pavillard [2]. Similarly, in this study, the IC_50_ value of irinotecan was found to be 5.4 mg/mL for 24 h. Irinotecan (CPT-11) requires metabolic activation in vivo to 7-ethyl-10-hydroxycamptothecin (SN-38) to exert its effects. SN-38 specifically interacts with DNA TOPO, disrupting the double-strand structure and ultimately triggering apoptosis [18,19]. Currently, CPT-11 serves as a primary chemotherapy option for colorectal cancer patients. However, its clinical use is often restricted due to severe side effects, such as delayed diarrhea [20]. A treatment strategy involving matrine combined with IR for colon cancer [21].

Beyond inducing apoptosis, the biological significance of acid ceramidase inhibition may extend to several key aspects of colorectal cancer progression. Dysfunctional sphingolipid metabolism is increasingly recognized as a feature of colorectal tumor formation, where elevated acid ceramidase activity shifts the ceramide/S1P balance toward cell survival, proliferation, inflammation, and treatment resistance. Therefore, pharmacological inhibition of acid ceramidase with Ceranib-2 can restore intracellular ceramide accumulation, suppress survival-promoting signaling, and sensitize tumor cells to chemotherapeutics. These mechanisms may partly explain the greater decrease in cell viability and increased apoptotic response observed following combination therapy with Irinotecan. Furthermore, modulation of sphingolipid metabolism has emerged as a promising strategy not only for inducing tumor cell death but also for overcoming drug resistance and enhancing the efficacy of conventional chemotherapy in colorectal cancer. 

The biological consequences of acid ceramidase inhibition extend beyond apoptosis induction. Ceramide is a centrally biologically active lipid that regulates numerous cellular processes involved in colorectal cancer progression, including cell proliferation, migration, epithelial plasticity, and cellular stress responses. Conversely, its downstream metabolite S1P promotes tumor cell survival, proliferation, angiogenesis, and inflammatory signaling. Therefore, inhibition of acid ceramidase with Ceranib-2 can shift the ceramide/S1P rheostat towards a tumor-suppressive state, which not only facilitates apoptotic cell death but also attenuates survival signaling pathways contributing to tumor progression and chemotherapy resistance [22]. Although the current study primarily evaluated cell viability and apoptosis, our findings support the hypothesis that modulation of sphingolipid metabolism may have broader biological effects, warranting further investigation in future studies, including those involving migration, invasion, epithelial-mesenchymal transition, and stress response signaling pathways.

Baspinar et al. investigated the effects of Ceranib-2 on Caco-2 colon cancer cells and reported that treatment with Ceranib-2 reduced cell viability in a dose- and time-dependent manner. Furthermore, Ceranib-2 significantly reduced TNFR1 mRNA expression while increasing ASAH gene expression, suggesting that inhibition of acid ceramidase modulates sphingolipid metabolism and promotes apoptosis signaling. Importantly, herein it was also demonstrated that the combination of Ceranib-2 and IR produced a greater antitumor effect than either treatment alone, supporting the potential therapeutic benefit of combining acid ceramidase inhibition with conventional chemotherapy [23].

Recent studies further support the idea that acid ceramidase (ASAH1) is an attractive therapeutic target in colorectal cancer. A comprehensive review by Afrin et al. highlighted that dysregulation of ceramide metabolism is a hallmark of many malignancies and that pharmacological inhibition of acid ceramidase promotes intracellular ceramide accumulation, shifting the sphingolipid rheostat towards apoptosis. Furthermore, it has been suggested that targeting ceramide-metabolizing enzymes is a promising strategy to enhance the efficacy of conventional anticancer therapies, particularly in combination therapy approaches. In colorectal cancer, increased ASAH1 expression has been shown to be associated with tumor progression, while inhibition of acid ceramidase has been shown to enhance the antitumor effects of chemotherapeutic agents and promote apoptosis. These observations support our findings that Ceranib-2, an acid ceramidase inhibitor, enhances the antitumor activity of IR, further highlighting the therapeutic potential of combining ceramide metabolism modulation with standard chemotherapy in colorectal cancer [24]. Espinoza and Snider, reviewing the existing literature, reported that ASAH1 expression is elevated in colorectal cancer, and that pharmacological inhibition of acid ceramidase promotes ceramide accumulation, suppresses tumor cell survival, and enhances apoptotic signaling. The authors also highlighted that Ceranib-2 demonstrates antitumor activity in various malignancies, including colorectal, lung, breast, prostate, glioma, and cervical cancers. Consistent with these observations, our results demonstrate that Ceranib-2 enhances the cytotoxic activity of IR in HT-29 colorectal cancer cells, suggesting that modulation of sphingolipid metabolism may improve the efficacy of conventional chemotherapy [22].

Several limitations of this study should be considered. First, all experiments were performed using a single colorectal cancer cell line (HT-29), which may not fully represent the molecular and phenotypic heterogeneity of colorectal cancer. Therefore, the observed therapeutic effects of the combination of Ceranib-2 with IR should be validated in additional colorectal cancer models with different genetic and molecular backgrounds. Second, the mechanistic conclusions of this study are primarily based on cell viability, Annexin V apoptosis assay, and morphological observations. While these approaches consistently demonstrate increased antitumor activity following combination therapy, complementary molecular assays evaluating apoptotic signaling pathways, molecules involved in ceramide metabolism, and cell cycle regulatory proteins have not been performed. Such assays would provide a more comprehensive understanding of the molecular mechanisms responsible for the observed biological effects. Future studies incorporating these approaches, along with research on normal colon epithelial cells and additional colorectal cancer cell lines, will further clarify the therapeutic potential and translational significance of Ceranib-2 in colorectal cancer. 

The findings of this study demonstrated that the combination of Irinotecan and Ceranib-2 exhibited a synergistic effect in suppressing the proliferation of colon cancer cells and inducing apoptosis. Compared to monotherapy with Irinotecan or Ceranib-2, the combined Irinotecan and Ceranib-2 treatment was more effective in inducing DNA damage, this combination also resulted in more severe effects, including DNA fragmentation, which in turn caused apoptosis. From these results, it was shown that the combination of Ceranib-2 and Irinotecan caused apoptosis in colon cancer cells by inhibiting ceramidase, one of the basic intracellular pathway enzymes, and DNA topoisomerase I enzymes involved in cell division, and this combination has the potential to be a new alternative drug candidate for cancer treatment after further investigations. Due to the high heterogeneity of colorectal cancer as a disease comprising numerous molecular and phenotypic subtypes that differ in biological behavior and response to treatment, in particular, epithelial–mesenchymal transition (EMT) has emerged as a key determinant of tumor progression, metastatic potential, and treatment resistance. Therefore, the response to acid ceramidase inhibition may vary depending on the molecular characteristics and epithelial-mesenchymal status of individual colorectal tumors; this underscores the need to validate our findings in additional colorectal cancer models representing different molecular subtypes [25].

In conclusion, the combination of Ceranib-2 and Irinotecan significantly reduced cell viability and increased apoptosis in HT-29 colorectal cancer cells, a more pronounced effect compared to either treatment administered alone. These findings suggest that acid ceramidase inhibition may enhance the antitumor activity of Irinotecan under in vitro conditions. However, further mechanistic investigations, validation in multiple colorectal cancer models, studies in normal colon epithelial cells, and in vivo experiments are needed to confirm the biological mechanisms and determine the translational potential of this combination.

## Figures and Tables

**Table 1:** IC_50_ values of Ceranib-2 and Irinotecan on HT-24 cells after 24 h treatment.

HT-29 Cells	IC_50_ Value
Ceranib-2	4.1 (μM)
Irinotecan	5.4 (mg/mL)

## Data Availability

All data generated or analyzed during this study are included in this published article.
